# Efficacy and Safety of Kanggan Mixture for Influenza: Protocol for a Multicenter Open-Label Randomized Controlled Trial

**DOI:** 10.2196/89891

**Published:** 2026-06-02

**Authors:** Yilan Gao, Yue Ge, Qian Wang, Zibo Sun, Shuo Dong, Yanfen Tang, Guicai Zhang, Xianmei Zhou, Hailang He

**Affiliations:** 1 Affiliated Hospital of Nanjing University of Chinese Medicine Nanjing, Jiangsu Province China; 2 Nantong Hospital of Traditional Chinese Medicine Nantong, Jiangsu Province China; 3 Yancheng Hospital of Traditional Chinese Medicine Yancheng, Jiangsu Province China; 4 Affiliated Hospital of Nanjing University of Chinese Medicine Department of Respiratory Medicine, Jiangsu Province Hospital of Chinese Medicine Nanjing, Jiangsu Province China

**Keywords:** kanggan mixture, influenza, traditional Chinese medicine, randomized controlled trial, protocol

## Abstract

**Background:**

Influenza, a highly contagious acute respiratory illness, causes annual seasonal epidemics worldwide, imposing a substantial public health burden. Although neuraminidase inhibitors, such as oseltamivir, can shorten symptom duration, their use is limited by antiviral resistance and adverse reactions. Kanggan mixture (KGM), an in-hospital traditional Chinese medicine preparation at Jiangsu Province Hospital of Chinese Medicine, has been widely used in clinical practice for influenza treatment, showing favorable effects on fever reduction and symptom improvement; however, high-quality comparative evidence on the efficacy and safety of KGM remains limited. This study is designed to address this gap by evaluating whether KGM is noninferior to oseltamivir for the treatment of influenza.

**Objective:**

This study aims to determine whether KGM is noninferior to oseltamivir in improving the clinical outcomes in patients with influenza and to assess the safety profile of KGM.

**Methods:**

This study is a multicenter, open-label, randomized controlled trial to be conducted in 3 tertiary grade A hospitals in China. Eligible participants with influenza will be randomly assigned in a 1:1 ratio to receive either KGM or oseltamivir for 5 days. A total of 184 participants will be enrolled, with 92 (50%) participants in each group. The primary outcome is time to clinical alleviation. Secondary outcomes include time to defervescence, time to onset of antipyretic effect, individual influenza symptom scores, and total influenza symptom scores. Adverse events will be monitored throughout the trial to evaluate the safety of KGM.

**Results:**

This study was funded in July 2024. Recruitment began in September 2025 and is expected to be completed in June 2026. The study results are expected to be published by December 2026.

**Conclusions:**

This protocol describes a multicenter randomized controlled trial designed to generate comparative evidence on the efficacy and safety of KGM for influenza treatment. The findings of this study are expected to provide initial clinical evidence supporting the use of KGM in the treatment of influenza, clarify its therapeutic value as a potential alternative to oseltamivir, and offer a novel alternative strategy for influenza management while contributing to the evidence base for integrating traditional Chinese medicine into influenza care.

**Trial Registration:**

International Traditional Medicine Clinical Trial Registry ITMCTR2025000743; https://itmctr.ccebtcm.org.cn/mgt/project/view/-5368318132552339899

**International Registered Report Identifier (IRRID):**

DERR1-10.2196/89891

## Introduction

Influenza is a highly contagious acute respiratory illness caused by the influenza virus. Seasonal epidemics occur worldwide every year, and unpredictable pandemics can arise, imposing a substantial public health burden [1]. In China, approximately 3.4 million medical visits are attributable to influenza-like illness annually, with the mean economic cost per outpatient episode ranging from RMB ¥464 (US $65) to RMB ¥1320 (US $185, and an estimated 88,100 deaths per year are linked to influenza-associated respiratory disease, accounting for 8.2% of all respiratory disease mortality [2]. Notably, influenza is a leading cause of hospitalization for pulmonary respiratory diseases and remains a significant health burden for older individuals, neonates, and children [3-5].

Globally, oseltamivir, a neuraminidase inhibitor, is one of the most widely prescribed small-molecule antiviral agents against influenza. By blocking the release of progeny virions from infected cells, it curtails the replication of both influenza A and influenza B viruses and is approved for patients aged 1 year or older. A meta-analysis demonstrated that oseltamivir accelerates symptom alleviation and decreases the incidence of lower respiratory tract complications and hospitalization in adults [6]. Despite the widespread clinical application of oseltamivir, antiviral resistance and adverse reactions remain unresolved challenges [7,8]. Therefore, the development of effective and accessible countermeasures against influenza is a global priority.

Traditional Chinese medicine (TCM), with centuries of clinical antiviral experience, offers alternative strategies. The evidence suggests that combining TCM with oseltamivir shortens fever duration, hastens symptom resolution, and reduces the length of stay [9]. An open-label trial further revealed that TCM monotherapy for H1N1 influenza achieved a fever reduction equivalent to that of oseltamivir [10]. Kanggan mixture (KGM), a TCM widely used in clinical practice, is effective at dispersing wind heat, clearing heat, and resolving toxins. Over the past 2 decades, it has been widely used for the treatment of influenza in clinical practice, demonstrating consistent benefits for fever reduction and symptom improvement. KGM was originally an empirical prescription by Professor Zhou Zhongying, a famous Chinese medicine practitioner. It became an in-hospital preparation at Jiangsu Province Hospital of Chinese Medicine in 1998 and was approved by the Jiangsu Food and Drug Administration in 2006 (approval number Z20060004). The formula is composed of 12 botanical drugs, including honeysuckle, forsythia, mint, Platycodon, bitter almond, *Bistortae isatis root, Dryopteris crassirhizoma*, *Cimicifuga*, *Chizonepeta*, *Ligusticum chuanxiong*, and licorice. A previous study demonstrated that compared with the control, KGM had an efficacy rate of 96.67% for acute viral upper respiratory infection and reduced the time to defervescence [11].

However, high-level evidence of the efficacy of KGM in the treatment of influenza is lacking, and its safety profile requires systematic evaluation. Therefore, we designed this prospective, open-label, randomized, parallel-controlled, noninferiority trial to compare the efficacy and safety of KGM and oseltamivir in adults with influenza.

Therefore, we designed this prospective, multicenter, open-label, randomized, parallel-group, noninferiority trial to compare the efficacy and safety of KGM with those of oseltamivir in adults with influenza. We hypothesize that KGM is noninferior to oseltamivir in shortening the time to clinical alleviation and may improve influenza-related clinical outcomes with an acceptable safety profile.

## Methods

### Trial Design

**Figure 1:**
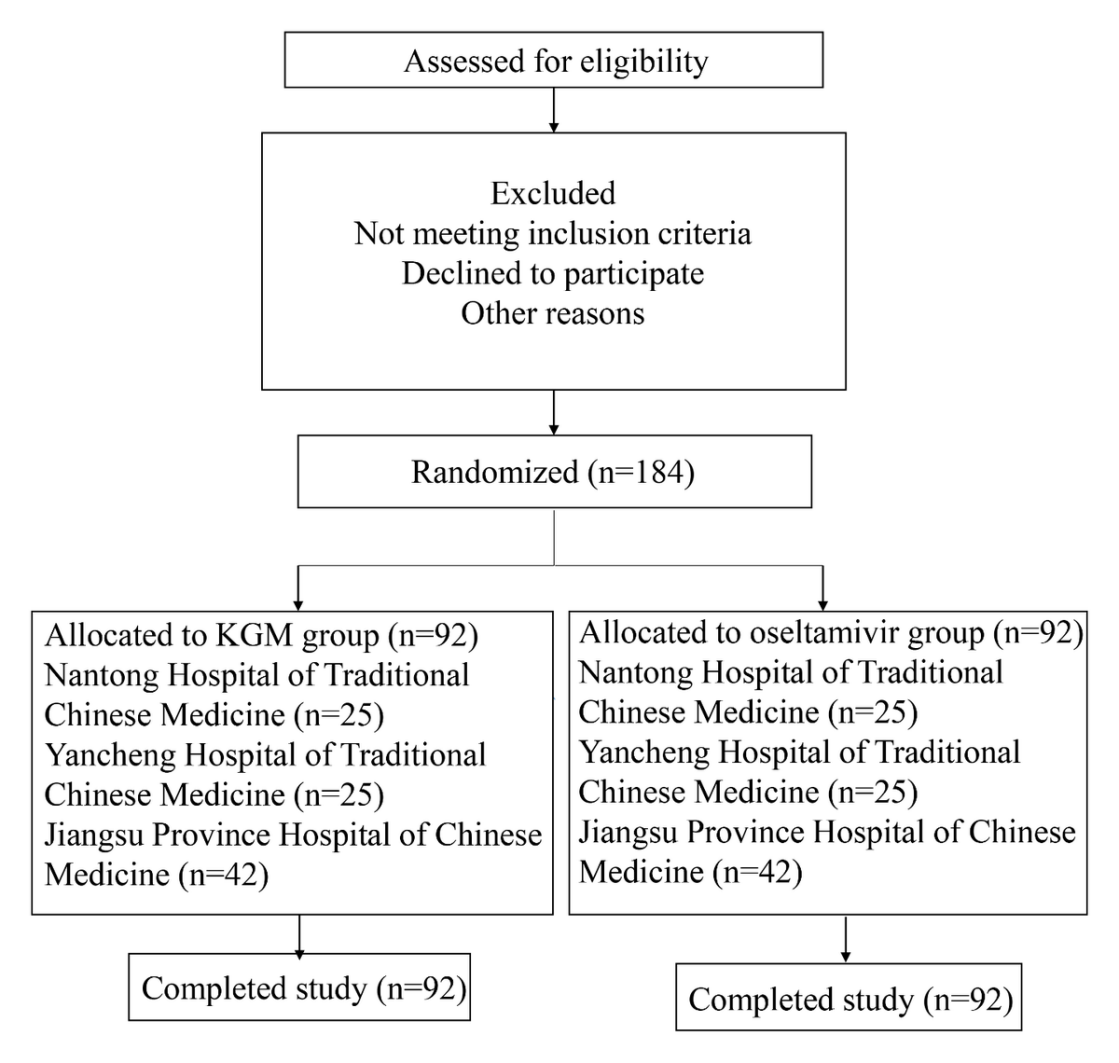
Flowchart of participant screening, randomization, allocation to the Kanggan Mixture (KGM) and oseltamivir groups, and study completion.

The completed SPIRIT (Standard Protocol Items: Recommendations for Interventional Trials) checklist is provided in Multimedia Appendix 1. This protocol describes an open-label, randomized controlled trial that will be conducted in accordance with the SPIRIT 2025 statement [12]. Eligible participants will be randomly allocated (1:1) to receive either KGM or oseltamivir for 5 consecutive days [13]. This study is designed as an open-label trial. Given the characteristics of the interventions, blinding of participants and treating physicians is not feasible. To address the potential risk of performance and detection bias associated with the open-label design, outcome assessors and statisticians will be blinded to treatment allocation whenever feasible; if blinding cannot be implemented, standardized outcome definitions, validated scoring tools, investigator training, and quality control procedures will be applied to minimize potential bias. The study will be conducted at the Jiangsu Provincial Hospital of Chinese Medicine, Nantong Hospital of Traditional Chinese Medicine, and Yancheng Hospital of Traditional Chinese Medicine. All the investigators will complete a standardized training program at Jiangsu Provincial Hospital of Chinese Medicine before patient enrollment. This training will include the study protocol, eligibility assessment, end point definitions, symptom scoring methods, case report form completion, and sample collection, processing, and storage procedures. In addition, before study initiation, the study start-up requirements and related study documents have already been unified across all participating centers. Standard operating procedures and uniform case report forms will be used across all centers. Eligibility criteria, end point definitions, symptom scoring methods, and sample collection, processing, and storage procedures will be standardized. Throughout the study, regular coordination meetings, on-site or remote monitoring, data verification, and query resolution processes will be conducted to oversee protocol adherence across centers. In addition, centralized data review and regular cross-center communication will be conducted to further enhance consistency across sites. If inconsistencies or protocol deviations are identified, retraining and corrective actions will be undertaken promptly to improve data homogeneity and minimize intercenter variability. This study was approved by the ethics committee of the Affiliated Hospital of Nanjing University of Chinese Medicine (2025-NL-002-02) and was prospectively registered with the International Traditional Medicine Clinical Trial Registry (ITMCTR2025000743). An independent Data Monitoring Committee was not established, as the study was considered to involve minimal risk. No interim analysis is planned for this trial. Early termination of the study will only occur in the event of serious safety concerns or ethical issues, such as unexpected adverse events (AEs) or protocol violations that could compromise participant welfare. Efficacy results will not be used as a basis for stopping the trial early. To ensure participant safety, an independent safety oversight plan has been implemented, with the study monitored by the institutional ethics committee and independent reviewers who are not involved in participant recruitment, treatment delivery, or outcome assessment. AEs and serious adverse events (SAEs) will be collected, reviewed periodically, and reported promptly to the principal investigator and ethics committee. If safety concerns arise, recommendations for protocol modification, temporary suspension, or early termination of the trial will be made. A flow diagram of the study design is provided in Figure 1.

### Sample Size

The sample size was determined based on the primary end point, namely the median time to the alleviation of influenza symptoms. Published studies have reported that the median time to symptom alleviation with oseltamivir is approximately 80 hours [14,15]. The assumption for KGM was informed by preliminary clinical evidence and prior clinical experience [11]. A noninferiority margin of 10 hours was prespecified with consideration of its clinical relevance and the feasibility of the trial. Under the assumptions of a 1:1 allocation ratio, a 2-sided significance level of .05, and 80% power, 78 evaluable participants were required per group. After accounting for an anticipated attrition rate of 15%, the target enrollment was set at 92 participants in each group, resulting in a total sample size of 184 participants.

### Recruitment

Participants will be recruited from 3 tertiary grade A hospitals in China. Recruitment strategies include screening outpatient and inpatient influenza cases, providing study information to potential participants via posters and hospital staff, and approaching eligible patients during routine clinical visits.

### Randomization and Allocation Concealment

Central stratified block randomization will be performed. All participant identifiers will be removed and replaced with study codes to ensure privacy protection. The allocation sequence was computer-generated via SAS 9.1 PROC PLAN (SAS Institute Inc) with a predefined seed. Sequentially numbered opaque sealed envelopes containing allocation codes will be centrally managed at each participating center.

### TCM Syndrome Differentiation

According to the 2016 Technical Guidelines for Clinical Research on TCM New Drugs for Influenza, wind heat invading the defensive *qi* is diagnosed when a patient presents with at least 2 major and 2 minor symptoms together with specified tongue and pulse findings.

The major symptoms include fever and sore throat. The minor symptoms include cough, nasal congestion or rhinorrhea, thirst, headache, myalgia, fatigue, and aversion to wind. Tongue and pulse findings include a red tongue body with a thin yellow or white coating and a floating and rapid pulse.

### Eligibility Criteria

Eligible participants must meet all inclusion criteria and none of the exclusion criteria to be enrolled in this study. The inclusion and exclusion criteria are presented in Textbox 1.

**Inclusion and exclusion criteria.**

**Inclusion**

- Meets the diagnostic criteria for influenza and TCM syndrome of “wind heat invading the defensive *qi”*
- Aged 18 to 65 years, any sex
- Symptom onset ≤48 hours prior to enrollment
- Able to complete all the study assessments
- Written informed consent is provided in accordance with the Good Clinical Practice

**Exclusion**

- Antiviral therapy was given for >48 hours prior to screening
- Severe renal impairment or the need for renal replacement therapy
- Altered mental status or seizures
- Severe gastrointestinal symptoms (intractable nausea, vomiting, diarrhea, or dehydration)
- Critical illness (respiratory failure, acute necrotizing encephalopathy, septic shock, multiorgan dysfunction, or the need for intensive care)
- Active tuberculosis, measles, AIDS, or other specified infectious diseases
- Pregnancy, breastfeeding, or within 2 weeks post partum
- Known hypersensitivity to oseltamivir or any study herbal component
- Participation in another anti-influenza trial within the past 28 days
- Investigator-judged inability to comply with study procedures
- A markedly elevated neutrophil count suggesting bacterial bronchitis

### Informed Consent

After confirming eligibility, the study physicians will explain the potential benefits and risks to the participants and their families. Adequate time will be provided for consideration and questioning. Written informed consent, including consent for the future use of biological samples (serum, plasma, urine, and stool), will be obtained in accordance with Good Clinical Practice guidelines. These biological samples will be used specifically for exploratory analyses directly related to the study objectives, including biomarker research, virological analysis, and pharmacokinetic analysis. The intended use of each sample type and any future analyses will be clearly explained to participants during the informed consent process. In addition, if participants wish to be informed of the study outcomes, a summary of the results will be provided to them. In line with international ethical standards, findings that involve major health concerns, have direct clinical applicability, or possess clear clinical validity will be communicated to participants both during and after the study. Conversely, information that does not meet standards of scientific validity or clinical significance will not be disclosed to participants to avoid unnecessary confusion or misinterpretation.

### Interventions

#### Choice of Comparators

KGM was selected as the experimental intervention on the basis of 20 years of clinical use in China, demonstrating efficacy in fever reduction and symptom improvement.

Preliminary safety and efficacy data are available. Oseltamivir (75 mg capsules, batch number H20213875) will be used as an active comparator.

#### Regimen

The intervention regimens are presented below.

- KGM: 50 mL orally twice daily, 30 minutes after meals, for 5 days (250 mL/bottle).
- Oseltamivir: 75 mg orally twice daily, 30 minutes after meals, for 5 days.

KGM was supplied by Jiangsu Provincial Hospital of Chinese Medicine, and oseltamivir was supplied by Sichuan Kelun Pharmaceutical Co, Ltd.

### Adherence and Concomitant Care

In addition to scheduled clinical observations on day 1, day 3, and 48 hours after the last dose, medication adherence will be monitored throughout the study. Participants will be instructed to record daily drug intake via a medication record card, and checks will be performed at each visit to verify compliance. Any discrepancies or missed doses will be documented in the case report form. During the study period, participants will not be permitted to use any other Chinese herbal medicines for the treatment of influenza. If participants develop symptoms such as high fever, headache, or sore throat, antipyretic and analgesic medications may be used concomitantly. These medications will be provided by the study sponsor.

### Outcomes

#### Overview

The assessment schedule is summarized in Table 1.

**Table 1:** Assessment schedule of the trial.

Assessments			Baseline (day 0)		Day 3		Day 5
**Eligibility and enrollment**							
	Inclusion criteria	✓					
	Exclusion criteria	✓					
	Informed consent	✓					
	Baseline assessment	✓					
	Randomization and allocation	✓					
**Primary outcome measurement**							
	Time to symptom relief	✓		✓		✓	
**Secondary outcome measurements**							
	Time to onset of antipyretic effect	✓		✓		✓	
	Duration of antipyretic effect	✓		✓		✓	
	Changes in viral load in nasopharyngeal secretions	✓				✓	
	Individual influenza symptom score	✓		✓		✓	
	Total influenza symptom score	✓		✓		✓	
	Changes in the traditional Chinese medicine syndrome score compared with baseline	✓				✓	
**Safety assessments**							
	Blood routine tests	✓				✓	
	Urine routine tests	✓				✓	
	Liver and renal function assessments	✓				✓	
	Electrocardiograms and chest radiography	✓				✓	
Adverse events			✓		✓		✓

#### Primary Outcome

The primary outcome is time to clinical alleviation, defined as the interval from the first dose to sustained clinical improvement, namely body temperature returning to normal, defined as an axillary temperature of ≤37 °C, and all remaining influenza symptom scores being 0 (absent) or 1 (mild) for at least 24 hours.

#### Secondary Outcomes

The secondary outcomes are as follows:

- Time to antipyretic onset: the interval from the first dose to the first documented decrease in axillary body temperature of at least 0.5 °C from baseline.
- Time to defervescence: time from the patient’s first dose until body temperature decreases to ≤37 °C and remains without recurrence for at least 24 hours.
- Changes in viral load in nasopharyngeal secretions: nasopharyngeal specimens will be collected at 2 predefined time points, day 0 (baseline) and day 5 (posttreatment), to evaluate changes in viral load over time. Viral load will be assessed using quantitative RT-PCR as a secondary virologic outcome to explore the effect of KGM on viral clearance.
- Individual influenza symptom scores: headache, fever or chills, myalgia, fatigue, cough, sore throat, and nasal congestion will each be scored on a 4-point ordinal scale ranging from 0 to 3, where 0 indicates absence of symptoms and 3 indicates severe symptoms.
- Total influenza symptom score: change from baseline and percentage change after dosing.

### Safety Assessments

No SAEs related to KGM have been reported, and systematic safety monitoring is planned. The parameters will include vital signs, complete blood count, urinalysis, hepatic and renal function tests, 12-lead electrocardiography, chest radiography, and AE surveillance.

### AE Management

All AEs will be recorded in the case report form, investigated thoroughly, and followed up until resolution or stabilization. Severity will be graded as mild, moderate, or severe. Follow-up modalities (inpatient care, outpatient visits, home visits, and telephone contacts) will be tailored to severity and clinical need. All SAEs will be reported to the ethics committee within 24 hours. Withdrawal criteria will include the following: (1) occurrence of an SAE related to the study intervention, (2) poor compliance with the study protocol, (3) participant request to withdraw, or (4) other medical conditions deemed inappropriate for continuation by the investigators.

### Statistical Methods

Study data will be entered into the electronic database independently by 2 trained research staff members, and discrepancies will be resolved by cross-checking to ensure accuracy. All data will be stored in a password-protected, access-restricted electronic database, with access limited to authorized study personnel.

Data management and analysis will be performed using SAS (version 9.4; SAS Institute) according to a prespecified statistical analysis plan. Statistical significance will be defined as a 2-sided *P* value of <.05, and 95% CIs will be reported.

Continuous variables will be summarized as mean (SD) for normally distributed data or median (IQR) for nonnormally distributed data. Categorical variables will be presented as frequencies and percentages. Normality will be assessed using the Shapiro-Wilk test, and the equality of variances will be examined using the Levene test. Between-group comparisons of continuous end point will be performed using an independent-sample 2-tailed *t* test (normal data) or the Mann-Whitney *U* test (nonnormal data). Categorical outcomes will be compared with the chi-square test or Fisher exact test, as appropriate. McNemar test will be performed to evaluate paired proportions when preintervention and postintervention data are available. All analyses will be conducted on both the full-analysis and per-protocol populations. For the primary outcome, missing data will be handled using multiple imputation, following current best practices, to reduce potential bias and improve the robustness of the findings. In addition, a sensitivity analysis will be performed using complete-case (listwise deletion) analysis to examine the consistency of the results under different missing-data assumptions.

### Auditing

An independent auditing mechanism will be established to ensure data integrity and protocol compliance. Auditing will be conducted at regular intervals by the institutional clinical research center, independently of the investigators, and audit reports will be submitted to the ethics committee.

### Ethical Considerations

This study was approved by the Ethics Committee of the Affiliated Hospital of Nanjing University of Chinese Medicine (approval number 2025-NL-002-02). Written informed consent will be obtained from all participants before enrollment in accordance with the Declaration of Helsinki and Good Clinical Practice guidelines, and the model consent form is provided in Multimedia Appendix 2.

After confirming eligibility, the study physicians will explain the potential benefits and risks of the study to participants and, when applicable, their family members. Adequate time will be provided for consideration and questions. Written informed consent, including consent for the future use of biological samples, including serum, plasma, urine, and stool, will be obtained in accordance with Good Clinical Practice guidelines. These biological samples will be used specifically for exploratory analyses directly related to the study objectives, including biomarker research, virological analysis, and pharmacokinetic analysis. The intended use of each sample type and any future analyses will be clearly explained to participants during the informed consent process.

If participants wish to be informed of the study outcomes, a summary of the results will be provided to them. In line with international ethical standards, findings that involve major health concerns, have direct clinical applicability, or possess clear clinical validity will be communicated to participants during and after the study. Conversely, information that does not meet standards of scientific validity or clinical significance will not be disclosed to participants to avoid unnecessary confusion or misinterpretation.

Any substantial protocol amendments will be submitted to the ethics committee and updated in the trial registry and relevant publications, as appropriate. To protect participant confidentiality, all study data will be deidentified before analysis. No compensation will be provided to participants. No additional posttrial care will be provided; participants will continue to receive standard medical care at their treating hospital.

## Results

This study was funded in July 2024. Participant recruitment commenced in September 2025 and is expected to be completed by June 2026. Any substantial protocol amendments will be submitted to the ethics committee for review and updated in the trial registry and relevant publications as appropriate. Completion of data analysis and publication of study results are anticipated by December 2026.

## Discussion

This study is expected to determine whether KGM is noninferior to oseltamivir in shortening the time to clinical alleviation of influenza-related symptoms in adults while also providing additional evidence on its safety profile. If the study hypothesis is confirmed, KGM may represent a clinically relevant treatment option for influenza symptom management.

KGM has been used for the treatment of influenza virus-related respiratory diseases for more than 20 years. However, high-quality comparative evidence from randomized controlled trials evaluating its efficacy and safety in adults with influenza remains limited. By using oseltamivir as an active comparator, this trial is designed to address an important evidence gap regarding the role of KGM in influenza management. The time to clinical alleviation was designated as the primary outcome, which is similar to that of a previous study conducted by Nong et al [16]. This outcome was selected because it is clinically meaningful and reflects recovery from the overall symptom burden of influenza.

This study has several strengths. First, this is a multicenter, randomized, controlled, noninferiority trial, and the multicenter design may improve the representativeness of participants from different regions and enhance the external validity of the findings. Second, oseltamivir was selected as the comparator because it is a standard antiviral treatment for influenza, thereby providing a clinically relevant benchmark for evaluating the therapeutic value of KGM. Third, standardized training, unified case report forms, prespecified outcome definitions, and centralized monitoring procedures are expected to improve consistency across study sites and strengthen data quality.

This trial has several limitations. First, some symptoms, such as cough, often persist for 1 to 2 weeks [17]. Therefore, the 5-day follow-up period may not fully capture the resolution of all influenza-related symptoms, particularly those that extend beyond the acute phase [18]. Second, the absence of a placebo control may limit the assessment of the absolute efficacy of KGM and may lead to potential overestimation of treatment effects. Third, because this is an open-label trial, performance bias and assessment bias cannot be fully excluded, particularly for subjective symptom outcomes, even though standardized outcome definitions, validated scoring tools, and quality control procedures will be used to minimize bias. These design characteristics may affect the interpretation of the observed effect size and should be carefully considered when interpreting the study findings [19,20].

If KGM is shown to be noninferior to oseltamivir with an acceptable safety profile, the study may support its use as an alternative therapeutic option for adults with influenza and provide a basis for future confirmatory studies in broader populations and settings. The study findings are planned to be disseminated through peer-reviewed publication and academic conference presentations, which may further inform clinical practice and future research on TCM for influenza.

In summary, this protocol describes a multicenter randomized controlled trial designed to generate comparative evidence on the efficacy and safety of KGM for influenza treatment. The findings of this study are expected to clarify the therapeutic value of KGM and contribute to the evidence base for influenza management while acknowledging the limitations related to the lack of placebo control and blinding, as well as their potential impact on effect size interpretation.

## Data Availability

The datasets generated and analyzed during this study will be available from the corresponding author upon reasonable request.

## Appendix

Multimedia Appendix 1 SPIRIT checklist.

Multimedia Appendix 2 Informed consent form for the multicenter, open-label, randomized controlled clinical study of Kanggan mixture for the treatment of influenza.

## Abbreviations

AE: adverse event
KGM: Kanggan mixture
SAE: serious adverse event
SPIRIT: Standard Protocol Items: Recommendations for Interventional Trials
TCM: traditional Chinese medicine
