# Supplementary material for: Efficacy and Safety of Kanggan Mixture for Influenza: Protocol for a Multicenter Open-Label Randomized Controlled Trial
Source: JMIR Res Protoc. 2026 Jun 2;15:e89891. doi: 10.2196/89891 (PMC13273194; doi:10.2196/89891)
Supplement: Multimedia Appendix 2 [file resprot_v15i1e89891_app2.pdf]

Informed Consent Form

Dear Participant,

You are invited to participate in the clinical research project titled "A Multicenter, Open-Label, Randomized Controlled Clinical Study on Kanggan Mixture for the Treatment of Influenza." This project is initiated by an open research grant approved by the Jiangsu Provincial Research Center for Infectious Diseases of Traditional Chinese Medicine. This is a prospective, multicenter, open-label, randomized controlled clinical study. The study will be conducted jointly at several hospitals, including the Affiliated Hospital of Nanjing University of Chinese Medicine (Jiangsu Provincial Hospital of Traditional Chinese Medicine), Nantong Hospital of Traditional Chinese Medicine, and Yancheng Hospital of Traditional Chinese Medicine. It is planned that the Affiliated Hospital of Nanjing University of Chinese Medicine will enroll 84 cases, while Nantong Hospital of Traditional Chinese Medicine and Yancheng Hospital of Traditional Chinese Medicine will each enroll 50 cases, with an estimated total of 184 participants volunteering for this study. This study has been reviewed and approved by the Ethics Committee of the Affiliated Hospital of Nanjing University of Chinese Medicine.

Before you decide whether to participate, please read the following information as carefully as possible. It is designed to help you understand the study, including why it is being conducted, why you are being asked to participate, the procedures and duration involved, and the potential benefits, risks, and discomforts associated with your participation. If you wish, you may discuss this information with your family or friends, or ask your physician to explain it to assist you in making an informed decision.

Why is this study being conducted?

Influenza is a highly contagious infectious disease worldwide, with its occurrence rate and mortality continuously rising, posing a significant threat to human health. Western medical treatment primarily relies on antiviral drugs, which carry potential risks of adverse reactions, and existing viral strains can mutate, leading to drug resistance. Kanggan Mixture is a hospital preparation of the Affiliated Hospital of Nanjing University of Chinese Medicine (Jiangsu Provincial Hospital of Traditional Chinese Medicine) and has been used clinically for over 20 years to treat influenza, demonstrating good therapeutic efficacy. This study aims to evaluate the clinical non-inferiority of Kanggan Mixture compared to oseltamivir in treating influenza with the TCM pattern of wind-heat invading the defensive level, as well as to assess the clinical safety of Kanggan Mixture in treating influenza.

The inclusion criteria for participants in this study are as follows:

1. Meet the Western medical diagnostic criteria for influenza.
2. Meet the Traditional Chinese Medicine (TCM) diagnostic criteria for the pattern of wind-heat invading the defensive level.
3. Aged between 18 and 65 years, with no gender restrictions.
4. Provide informed consent voluntarily to participate. The process of obtaining informed consent must comply with Good Clinical Practice (GCP) guidelines.

What Does Participation in the Study Involve?

1. The title of this research project is "A Multicenter, Open-Label, Randomized Controlled Clinical Study on Kanggan Heji for the Treatment of Influenza." The control group in this study will receive Oseltamivir, administered orally at a dose of 75 mg twice daily. The Oseltamivir is produced by Sichuan Kelun Pharmaceutical Co., Ltd., with

specifications of 75 mg per capsule and batch number H20213875. The treatment group will receive Kanggan Heji, administered orally at a dose of 50 ml twice daily. The Kanggan Heji is supplied by Jiangsu Provincial Hospital of Traditional Chinese Medicine, with specifications of 250 ml per bottle and batch number Z20060004.

2. Prior to your participation in this study, your medical history will be inquired about and recorded, and a physical examination will be conducted to determine your eligibility for participation.

3. If you meet the inclusion and exclusion criteria, the study will proceed as follows: At the beginning of the study, you will be assigned to either the treatment group (Kanggan Heji) or the control group (Oseltamivir) based on a computer-generated random number. Participants have a 50% chance of being assigned to either group. The treatment duration with Kanggan Heji is 5 days. The following parameters will be assessed by the physician: body temperature, respiratory rate, heart rate, cardiac rhythm, blood pressure, complete blood count, urinalysis, stool routine test, liver function (Alanine Aminotransferase [ALT], Aspartate Aminotransferase [AST]), renal function (Urea, Creatinine), and electrocardiogram (ECG).

4. This study requires the observation and recording of your primary symptoms and signs on the first day of the initial visit, day 3, and within 48 hours after the last dose. Laboratory tests will be performed once before and once after the treatment (a re-examination of liver and kidney function will be conducted within 7 days after the end of treatment).

5.Medication and Other Considerations： You must take the medication strictly according to the doctor's instructions. During the study period, you are not permitted to use any other Chinese herbal medicines for the treatment of influenza. If you develop symptoms such as high fever, headache, or sore throat, antipyretic and analgesic medications may be used concomitantly; these will be provided by the study sponsor. Should you require any other treatments, please contact your physician in advance.

Who Should Not Participate in This Study?

1. Participants who have received antiviral treatment for influenza infection for more than 48 hours prior to screening.
2. Participants with known severe renal impairment or those undergoing continuous renal replacement therapy, hemodialysis, or peritoneal dialysis.
3. Participants presenting with mental status changes or convulsions (e.g., slowed response, drowsiness, and restlessness).
4. Participants experiencing severe gastrointestinal symptoms (e.g., severe nausea, vomiting, diarrhea, or even dehydration).
5. Participants with any of the following critical illnesses: respiratory failure, acute necrotizing encephalopathy, septic shock, multiple organ dysfunction, or other severe clinical conditions requiring intensive care.
6. Participants suffering from tuberculosis, measles, AIDS, or other infectious diseases.
7. Pregnant women (including those with a positive pregnancy test at enrollment), women who are breastfeeding, or women within 2 weeks postpartum.
8. Participants determined by the investigator to have a history of allergy or severe intolerance to oseltamivir or herbal medicines.
9. Participants who are currently participating or have participated in another anti-influenza treatment trial within

the past 28 days.

10. Participants deemed by the investigator as unlikely to comply with study visits, self-assessments, and interventions.

What are the potential risks of participating in the study?

All investigational drugs may cause side effects, and adverse reactions such as nausea, vomiting, and diarrhea may occur. If any adverse reaction arises during the study, regardless of its relationship to the drug, the physician will assess the condition and provide appropriate medical treatment. Every effort will be made to prevent and manage any harm potentially resulting from participation in this study. In the event of an adverse event during the clinical trial, the investigator will determine whether it is related to the investigational drug. For injuries clearly associated with the investigational drug, the sponsor will cover the corresponding medical expenses, as stipulated in China's Good Clinical Practice guidelines.

What are the potential benefits of participating in the study?

Participation in this study may lead to an improvement in your condition. Additionally, this research will help determine which treatment approach is safer and more effective for other patients with conditions similar to yours.

Are there any costs associated with participating in the study?

The sponsor of this study will cover all study-related examination costs during the research period and will provide the investigational drug free of charge. Covered examinations include pregnancy tests, posteroanterior chest X-rays, etiological tests, influenza virus antigen detection via test kit, nasopharyngeal secretion influenza virus nucleic acid testing, complete blood count, liver and kidney function tests, and electrocardiograms. Should any injury related to the investigational drug occur, the sponsor will also assume the corresponding medical expenses. However, treatments or examinations required for other concurrent medical conditions are not included.

Confidentiality of Personal Information:Your medical records will be stored at the hospital. The investigators, relevant regulatory authorities, and the ethics committee will be permitted access to your medical records. Any public reports resulting from this research will not disclose your personal identity. We will make every effort to protect the privacy of your personal medical information within the limits of the law.

Notification of Research Results:If you wish to be informed of the research results, please inform us. We will record your request and provide you with the study findings upon its completion. According to international ethical guidelines, data concerning significant health issues, information with direct clinical applicability, or findings with direct clinical validity will be disclosed to research participants, either during or after the study. Conversely, information lacking scientific validity and clinical importance is not suitable for communication with research participants.

Voluntary Participation:Participation in this study is entirely voluntary. You may refuse to participate or withdraw from the study at any time without affecting your medical care. If you choose not to participate or decide to withdraw, numerous alternative treatments are available, such as baloxavir marboxil. Should you decide to withdraw from the study, please contact your physician. You may be required to undergo relevant examinations, which would be beneficial for protecting your health.

Will my medical records and biospecimens be used for research other than this study? In accordance with relevant

national regulations, any future use or continued storage of remaining specimens after the clinical trial must be addressed in the informed consent form signed by the participant. This includes specifying the duration of storage, measures for data confidentiality, and the conditions under which data and samples may be shared with other researchers. Therefore, your medical records and biospecimens will not be used for other studies without your informed consent.

Participant’s Declaration: I have read the above information regarding this study and fully understand the potential risks and benefits associated with participation. I voluntarily agree to take part in this study.

Subject Signature: ________ Date: ____ Year ____ Month ____ Day

Subject's Contact Telephone Number:Mobile:

Investigator's Declaration:I confirm that I have explained the details of this study to the subject, particularly the potential risks and benefits associated with participation.

Investigator Signature:________ Date: ____ Year ____ Month ____ Day

Investigator's Work Telephone Number: Mobile:

If you have any medical questions related to this study, please contact the investigator listed above. If you wish to express any dissatisfaction or concerns regarding your participation in this study, or if your personal rights have been infringed upon, please contact the Ethics Committee of the Affiliated Hospital of Nanjing University of Chinese Medicine at telephone number: 025-86560515.
